# Fragility fracture of talar neck in an osteoporotic patient treated by posterior-to-anterior screw fixation under hindfoot endoscopy: a case report

**DOI:** 10.1093/jscr/rjad029

**Published:** 2023-02-02

**Authors:** Futoshi Morio, Shota Morimoto, Toshiya Tachibana, Tomoya Iseki

**Affiliations:** Department of Orthopaedic Surgery, Hyogo Medical University, Nishinomiya, Hyogo, Japan; Department of Orthopaedic Surgery, Hyogo Medical University, Nishinomiya, Hyogo, Japan; Department of Orthopaedic Surgery, Hyogo Medical University, Nishinomiya, Hyogo, Japan; Department of Orthopaedic Surgery, Hyogo Medical University, Nishinomiya, Hyogo, Japan

## Abstract

Fragility fractures of the talar neck are extremely rare. Here, we describe a case of fragility fracture of the talar neck associated with osteoporosis in a 76-year-old female, who was treated by posterior-to-anterior screw fixation under hindfoot endoscopy. A 76-year-old female cleaner with a history of osteoporosis complained of pain in her right ankle when going downstairs. Radiological findings revealed a fragility fracture of the talar neck associated with osteoporosis. Because the patient was elderly and it was difficult to treat using a prolonged non-weight-bearing cast, we performed a posterior-to-anterior parallel dual screw fixation under hindfoot endoscopy for this case. As a result, the patient was able to return to work 8 weeks after surgery without pain, dysfunction or complication. Osteosynthesis with posterior-to-anterior screw fixation under hindfoot endoscopy successfully treated a rare case of fragility fracture of the talar neck in a 76-year-old female cleaner.

## INTRODUCTION

The talar neck is the most common site, accounting for 50% of all talar fractures [1, 2]. Fractures of the talar neck generally occur by high-energy trauma, such as falling from a great height and traffic accidents [3]. In general, fragility fractures are caused by the repetition of low-energy trauma and are often associated with osteoporosis [4].

In terms of surgical treatment for talar neck fractures, a screw is generally inserted from the anterior part of the talus [5, 6]; insertion from the posterior section of the talus has biomechanical and anatomical advantages over anterior insertion in that it provides greater fixation and avoids chondral injury to the talonavicular joint [7, 8]. However, there have been reported risks to this approach, such as injury to the sural nerve and flexor hallucis longus (FHL), owing to a narrower safety zone for screw insertion [9].

A two-portal hindfoot endoscopic technique, described by van Dijk *et al.* [10] in 2000, allows direct visualization of the hindfoot structures. To the best of our knowledge, there are no reports of a posterior-to-anterior screw fixation used to treat a fragility fracture of the talar neck under hindfoot endoscopy.

In this report, we describe a rare case of a talar neck fragility fracture in a 76-year-old female with osteoporosis, which was treated by endoscopic posterior-to-anterior screw fixation of the hindfoot. Written informed consent was obtained from the patient before publishing this report.

## CASE REPORT

**Figure 1:**
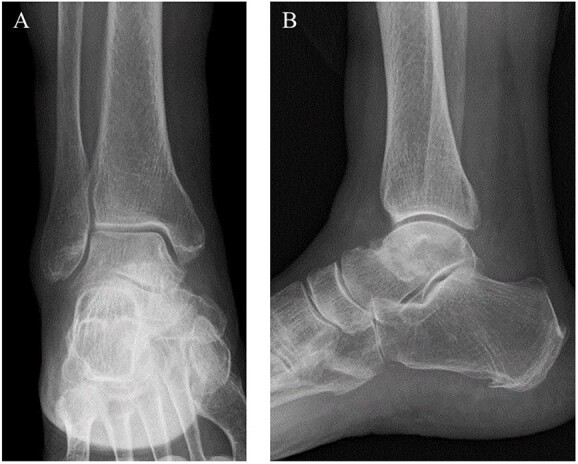
Preoperative plain radiographs showed no obvious abnormal findings from the (**A**) anteroposterior and (**B**) lateral views.

**Figure 2:**
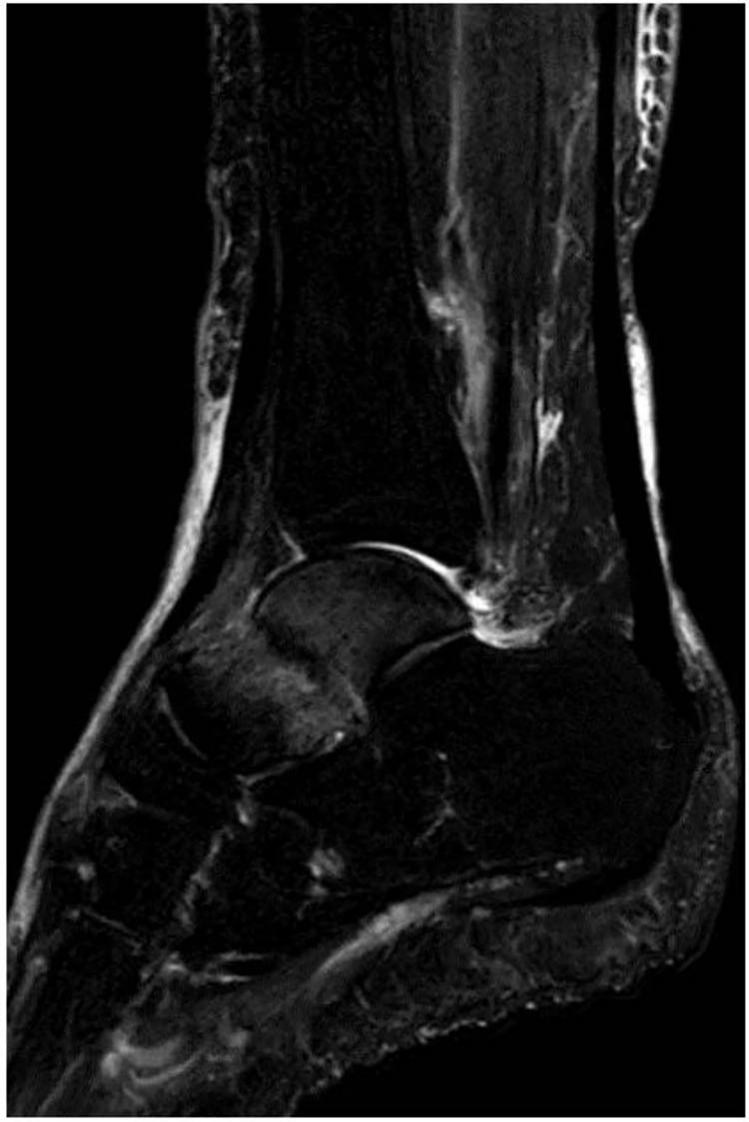
MRI revealed a low signal linear line in the talar neck and a bone marrow edema around the line on a fat-suppressed T2-weighted (FS-T2) image.

**Figure 3:**
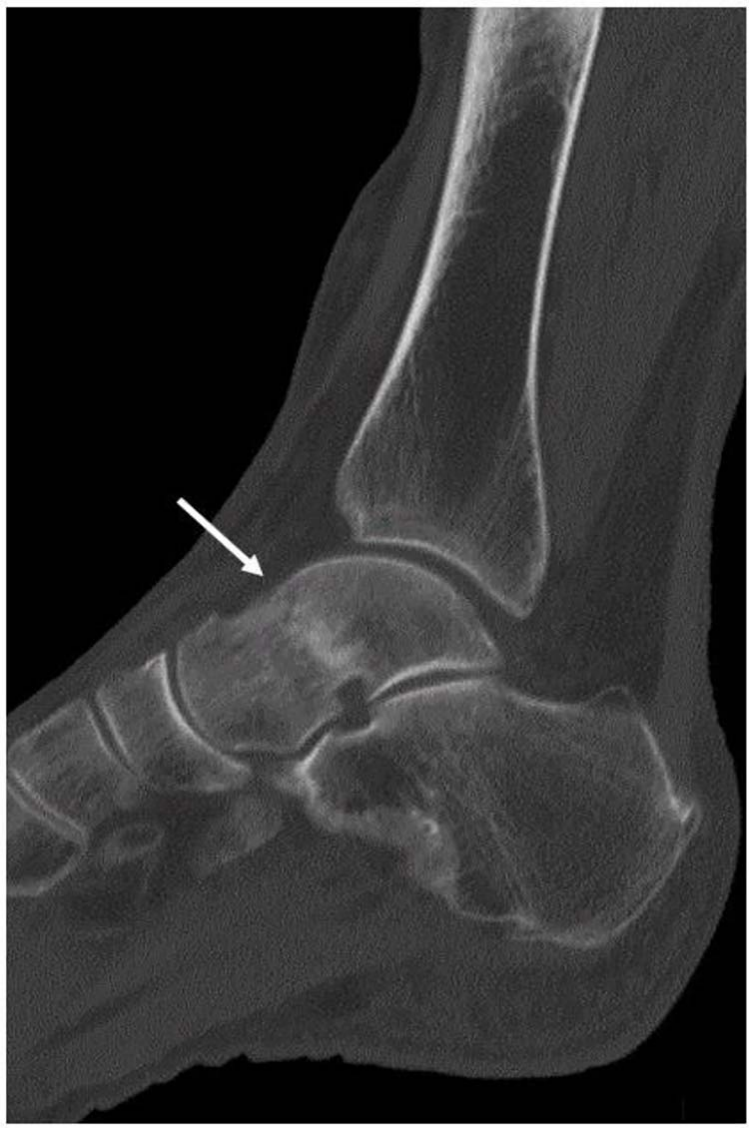
Although a non-contrast CT scan of the sagittal view demonstrated a fracture line in the talar neck (arrow), the bone fragment was not dislocated.

**Figure 4:**
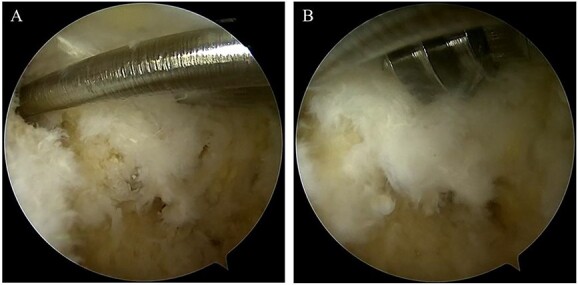
(**A**) After confirming the posterior part of the talar body and the FHL, two 1.6-mm diameter guidewires were parallelly inserted from the posterior part of the talar body to the talar head via under hindfoot endoscopy and fluoroscopy; (**B**) two cannulated, 4.5-mm diameter double-threaded screws were inserted through the guidewires.

**Figure 5:**
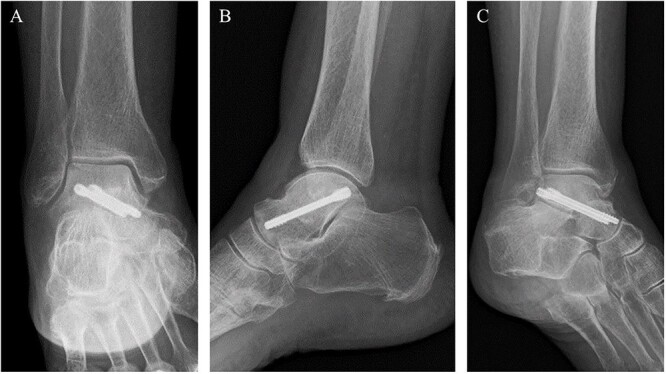
Post-operative plain radiographs revealed that two cannulated 4.5-mm double-threaded screws were inserted from the posterior part of the talus to the talar head as seen from the (**A**) anteroposterior, (**B**) lateral and (**C**) oblique views.

A 76-year-old female, who worked as a cleaner, complained of pain in her right ankle when going downstairs while working. She had medical history of only osteoporosis and had taking daily active vitamin D for >10 years. She was given conservative treatment for 1 month at a local clinic, however, her ankle pain persisted and so she visited our clinic. Physical examinations revealed tenderness and swelling at the anterior aspect of the ankle. Plain radiographs showed no obvious abnormal findings (Fig. 1). Magnetic resonance imaging (MRI) revealed a low signal linear line in the talar neck and a bone marrow edema around the line (Fig. 2). Non-contrast computed tomography (CT) demonstrated an obvious fracture line in the talar neck, however, the bone fragment was not displaced (Fig. 3). Based on medical histories, clinical and radiological findings, we diagnosed her with a fragility fracture of the talar neck associated with osteoporosis. Because the patient was elderly and it was difficult to treat using a prolonged non-weightbearing cast, we applied operative treatment to allow early rehabilitation. The operation was performed under spinal anesthesia in a prone position with an air tourniquet and a fluoroscopy. The posteromedial and posterolateral portals were created according to van Dijk *et al.* [10]. First, the posterior aspect of the talus was observed using a 4.0-mm diameter 30° arthroscope, and soft tissues around the talus (including synovium and adipose tissues) were removed with a 3.5-mm diameter motorized shaver. After confirming the posterior part of the talar body and the FHL, two 1.6-mm diameter guidewires were parallelly inserted from the posterior part of the talar body to the talar head by hindfoot endoscopy and fluoroscopy (Fig. 4A), and two cannulated 4.5-mm diameter double-threaded screws (Double Thread Screw, Meira, Nagoya, Japan) were inserted through the guidewires (Fig. 4B). The wound was sutured, and the operation was concluded (Fig. 5). Active range of motion exercises were allowed immediately after surgery, and a non-weightbearing short leg splint was worn for 1 week. Partial-weight bearing was permitted at 2 weeks and full-weight bearing was permitted at 4 weeks post-operatively. In addition, daily injections of teriparatide (Forteo, Eli Lilly and Company, Indianapolis, IN, USA) were introduced 2 weeks post-operatively. Eight weeks after the operation, the patient was able to return to work without pain or functional impairment. One year postsurgery, the patient was still working as a cleaner without any symptoms or complications.

## DISCUSSION

The most common mechanism of talar neck fracture is reported to be contact of the talar neck with the anterior edge of the distal tibia due to hyperdorsiflexion of the talocrural joint in high-energy trauma [3]. Previous biomechanical studies have demonstrated that the talocrural joint motion, including dorsiflexion and plantarflexion, was highest when walking downstairs [11, 12]. We attributed the cause of the fragility fracture in this case to the poor bone quality of the talar neck and hyperdorsiflexion of the talocrural joint when walking downstairs.

Fan *et al*. [8] have reported that the biomechanical efficiency of posterior-to-anterior parallel dual screw fixation provided better results in the stress peak, stress distribution and displacement of the talus and internal fixation. In addition, posterior-to-anterior screw fixation has an anatomic advantage of avoiding damage to the talonavicular joint.

However, posterior-to-anterior screw fixation has anatomic risks, such as sural nerve and FHL damage, due to a narrower safety zone for screw insertion [13, 14]. A two-portal hindfoot endoscopic technique provides a direct and better visualization of the hindfoot structures and is associated with fewer complication rates [11]. A very low rate of sural nerve and FHL injury has been reported with the hindfoot endoscopic technique [15]. The rates of the sural nerve and FHL injury in this report were sufficiently low when compared to the percutaneous posterior-to-anterior screw fixation for fractures of the talar neck.

In this case, it was necessary to perform a posterior-to-anterior parallel dual screw fixation in order to obtain stronger fixation. However, insertion screws in the posterior part of the talus can pose such risks such as sural nerve and FHL injuries. Therefore, in order to avoid these risks, we performed a posterior-to-anterior parallel dual screw fixation under hindfoot endoscopy for this case. As a result, the patient was able to return to work 8 weeks after surgery without pain, dysfunction or complication.

In conclusion, we reported a rare case of fragility fracture of the talar neck associated with osteoporosis in a 76-year-old female, who was successfully treated by a safe and minimally invasive posterior-to-anterior screw fixation under hindfoot endoscopy. This surgical technique may be a useful option in the treatment of fragility fractures of the talar neck.

## Data Availability

Not applicable.

## References

[1] Junge T , Bellamy J, Dowd T, Osborn P. Outcomes of talus fractures associated with high-energy combat trauma. Foot Ankle Int 2017;38:1357–61. 28931325 10.1177/1071100717729124

[2] Fleuriau Chateau PB , Brokaw DS, Jelen BA, Scheid DK, Weber TG. Plate fixation of talar neck fractures: preliminary review of a new technique in twenty-three patients. J Orthop Trauma 2002;16:213–9. 11927801 10.1097/00005131-200204000-00001

[3] Jordan RK , Bafna KR, Liu J, Ebraheim NA. Complications of talar neck fractures by Hawkins classification: a systematic review. J Foot Ankle Surg 2017;56:817–21. 28633784 10.1053/j.jfas.2017.04.013

[4] Antoniadou E , Kouzelis A, Diamantakis F, Bavelou A, Panagiotopoulos E. Characteristics and diagnostic workup of the patient at risk to sustain fragility fracture. Injury 2017;48:17–23. 10.1016/j.injury.2017.08.033 28855082

[5] Capelle JH , Couch CG, Wells MW, Morris RP, Buford WL Jr, Merriman DJ, et al. Fixation strength of anteriorly inserted headless screws for talar neck fractures. Foot Ankle Int 2013;34:1012–6. 23456083 10.1177/1071100713479586

[6] Xue Y , Zhang H, Pei F, Chongdi T, Song Y, Fang Y, et al. Treatment of displaced talar neck fractures using delayed procedures of plate fixation through dual approaches. Int Orthop 2014;38:149–54. 24297608 10.1007/s00264-013-2164-2 PMC3890131

[7] Swanson TV , Bray TJ, Holmes GB. Fractures of the talar neck. A mechanical study of fixation. J Bone Joint Surg Am 1992;74:544–51. 1583049

[8] Fan Z , Ma J, Chen J, Yang B, Wang Y, Bai H, et al. Biomechanical efficacy of four different dual screws fixations in treatment of talus neck fracture: a three-dimensional finite element analysis. J Orthop Surg Res 2020;15:45–52. 32046746 10.1186/s13018-020-1560-8 PMC7014601

[9] Attiah M , Sanders DW, Valdivia G, Cooper I, Ferreira L, MacLeod MD, et al. Comminuted talar neck fractures: a mechanical comparison of fixation techniques. J Orthop Trauma 2007;21:47–51. 17211269 10.1097/01.bot.0000247077.02301.d0

[10] van Dijk CN , Scholten PE, Krips R. A 2-portal endoscopic approach for diagnosis and treatment of posterior ankle pathology. Art Ther 2000;16:871–6. 10.1053/jars.2000.19430 11078550

[11] Kuni B , Wolf SI, Zeifang F, Thomsen M. Foot kinematics in walking on a level surface and on stairs in patients with hallux rigidus before and after cheilectomy. J Foot Ankle Res 2014;7:13–22. 24524773 10.1186/1757-1146-7-13 PMC3925775

[12] Protopapadaki A , Drechsler WI, Cramp MC, Coutts FJ, Scott OM. Hip, knee, ankle kinematics and kinetics during stairs ascent and descent in healthy young individuals. Clin Biomech 2007;22:203–10. 10.1016/j.clinbiomech.2006.09.010 17126461

[13] Roberts LE , Pinto M, Staggers JR, Godoy-Santos A, Shah A, de Cesar NC. Soft tissue structures at risk with percutaneous posterior to anterior screw fixation of the talar neck. Foot Ankle Int 2018;39:1237–41. 29860866 10.1177/1071100718777771

[14] Beltran MJ , Mitchell PM, Collinge CA. Posterior to anteriorly directed screws for management of talar neck fractures. Foot Ankle Int 2016;37:1130–6. 27340258 10.1177/1071100716655434

[15] Donnenwerth MP , Roukis TS. The incidence of complications after posterior hindfoot endoscopy. Art Ther 2013;29:2049–54. 10.1016/j.arthro.2013.08.036 24286803

